# The role of bladder diverticula in the prevalence of acute urinary retention in patients with BPH who are candidates to surgery

**DOI:** 10.1590/S1677-5538.IBJU.2017.0605

**Published:** 2018

**Authors:** Alexandre Iscaife, Gabriel dos Anjos, Cristovão Barbosa, Willian Carlos Nahas, Miguel Srougi, Alberto Azoubel Antunes

**Affiliations:** 1 Divisão de Urologia, Faculdade de Medicina da Universidade de São Paulo - FMUSP, SP, Brasil

**Keywords:** Bladder Diverticulum [Supplementary Concept], Prostatic Hyperplasia, Urinary Retention

## Abstract

**Introduction:**

The urinary bladder diverticula (BD) secondary to benign prostatic hyperplasia (BPH) is a complication that can lead to urinary stasis, stone, urinary tract infection (UTI) and tumors. It's role in acute urinary retention (AUR) is not totally understood.

**Objectives:**

To determine the effect of BD size on AUR rates in patients with BPH candidates to surgery.

**Subjects and Methods:**

We performed a retrospective cohort study of 47 patients with BPH and BD who underwent BPH surgery associated to complete bladder diverticulectomy from 2006 to 2016. We analyzed risk factors for AUR in patients with BD using univariate, multivariate and correlation analysis.

**Results:**

There was a difference in the size of the diverticula, with 6.8 cm vs. 4.5 cm among patients with and without AUR respectively (p=0.005). The ROC curve showed a correlation between the size of BD and the risk of AUR. The value of 5.15 cm presented a sensitivity of 73% and a specificity of 72%. The area under the curve was 0.75 (p=0.01). Comparing groups with BD >5.0 cm vs. ≤5.0 cm, the AUR incidence was 74% and 27.8% respectively with an OR of 2.65 (1.20-5.85) (p=0.005). In the multivariate analysis, only the size of the diverticula reached statistical significance (p=0.012).

**Conclusions:**

The diameter of BD is an independent risk factor for AUR in patients with BPH and BD who are candidates to surgery. A diameter greater than 5.15 cm increases the risk of AUR.

## INTRODUCTION

The bladder diverticula (BD) secondary to benign prostatic hyperplasia (BPH) is more common in elderly men with an incidence range from 1 to 8% (1). Most of them are asymptomatic and discovered incidentally during investigation for other causes as BPH. The urine stasis in the diverticula can lead to complications such as stone formation, urinary tract infection (UTI) and tumors. The incidence of tumors in the diverticula range from 2 to 10% and due to the lack of the smooth muscle layer, the prognosis is usually worst (2, 3).

Publications about acquired BD are scarce in the contemporary urology literature and some authors consider it as consequence of advances in the treatment of BPH (2). In the other hand, the widespread use of medical treatment for BPH in the last 20 years may have caused an elevated incidence of BD due to an increase of bladder damage secondary to longer periods of obstruction.

Most of the BD are treated with observation and the classical indications for surgery includes the presence of tumor, persistent infection, bladder stone, ureteral obstruction, severe reflux of the diverticulum and retention due to high residue in the diverticula. However, the role of BD in the pathogenesis of LUTS is poorly understood (4).

To our knowledge, there are no studies analyzing the relationship between the size of a diverticula and the impairment on the bladder function. This analysis could help to define a cutoff value to indicate prostate surgery and diverticulectomy. In the present study, we determined the effect of BD diameter on acute urinary retention (AUR) rates.

## SUBJECTS AND METHODS

This is a retrospective cohort study of 47 patients who underwent BPH surgery and were submitted to complete bladder diverticulectomy (29 open surgeries and 18 laparoscopic surgeries) from May of 2006 to May of 2016. We used a database of surgical patients from the main Hospital of the University of Sao Paulo, a reference center in São Paulo – Brazil. The study was approved by the Ethical Committee under the number 5097. Patients with previous prostate or bladder surgery or suspected for congenital or iatrogenic bladder diverticula were not considered for analysis. All patients selected presented severe symptoms refractory to oral medications (alpha-blockers and 5 alpha-reductase inhibitors) and were candidates for prostate surgery.

**Figure 1:**
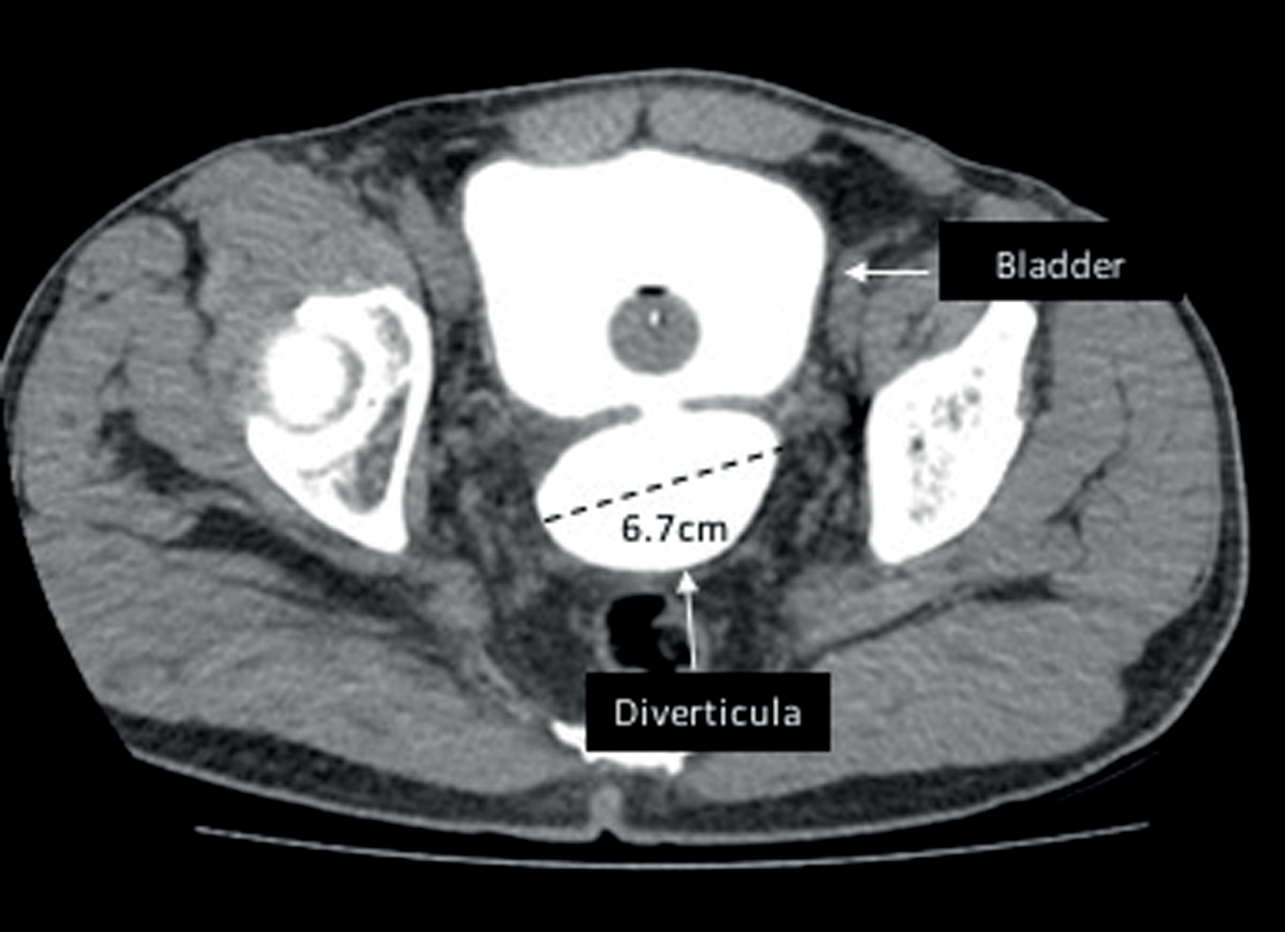
Diverticula assessment. pelvic computed Tomography (CT) with the bladder full of contrast showing posterior diverticula with 6.7cm and a narrow neck.

We compared age, diabetes mellitus (DM), hypertension, prostate weight, prostate specific antigen (PSA), uroflowmetry rates between groups with and without AUR. The effect of the diameter of bladder diverticula on these clinical characteristics and on the risk of AUR were also analyzed. The size of BD was evaluated through computed tomography (CT) with the bladder full of contrast (Figure-1).

The statistical analysis was performed with the SPSS 21.0 software using Student's t test for homogeneous variables, Mann-Whitney U test for non-homogeneous variables and Qui squared test for categorical variables. A correlation analysis with a ROC curve and a multivariated analysis for confounding variables were performed. For all statistical analysis, we considered a level of significance of 5% (P <0.05).

## RESULTS

Table-1 compares patients with BD with or without AUR. Average age was similar between groups. The prostatic weight was 70.7 g (±58.2) vs. 50.1g (±30.2) (p=0.19) and the PSA was 9.6 ng/dL (±21.0) vs. 3.5 ng/dL (±5.5) (p=0.22) comparing patients with and without AUR respectively. The pre and post- maximum flow rate (Qmax) were similar between groups. There was a difference in size of the diverticula, with 6.8 cm (±2.5) vs. 4.5 cm (±2.0) (p=0.005) in patients with and without AUR, respectively. The prevalence of hypertension was 40% in patients with AUR and 27.3% without AUR (p=0.35) and DM was 20% vs. 9.1% (p=0.29). The logistic regression with multivariate analysis for AUR as the dependent variable included age, hypertension, diabetes, prostate weight, PSA and only the size of the diverticula reached statistical significance with p=0.012.

**Table 1:** Data comparing patients with bladder diverticula with and without AUR.

			Univariate	Multivariate
	AUR+(n=25)	AUR-(n=22)	*p*	*p*
	Mean±SD	Mean±SD		
Age (years)	67.2 (12.7)	68.5 (10.4)	0.70 a	0.70
Prostate weight (g)	70.7 (58.2)	50.1 (30.2)	0.18 a	0.17
PSA (ng/dL)	9.6 (21.0)*	3.5 (5.5)*	0.22 b	0.70
Qmax pre (mL/s)	7.9 (7.1)	6.1 (4.9)	0.47 a	-
Qmax pos(mL/s)	24.8 (13.8)	19.6 (9.0)	0.20 a	-
Size of BD (cm)	6.8 (2.5)	4.5 (2.0)	0.005 a	0.01
Hypertension	40.0%	27.3%	0.35 c	0.42
Diabetes	20.0%	9.1%	0.29 c	0.42

a Student T Test;

b Mann Whitney;

c Chi Square

* median

**Figure 2:**
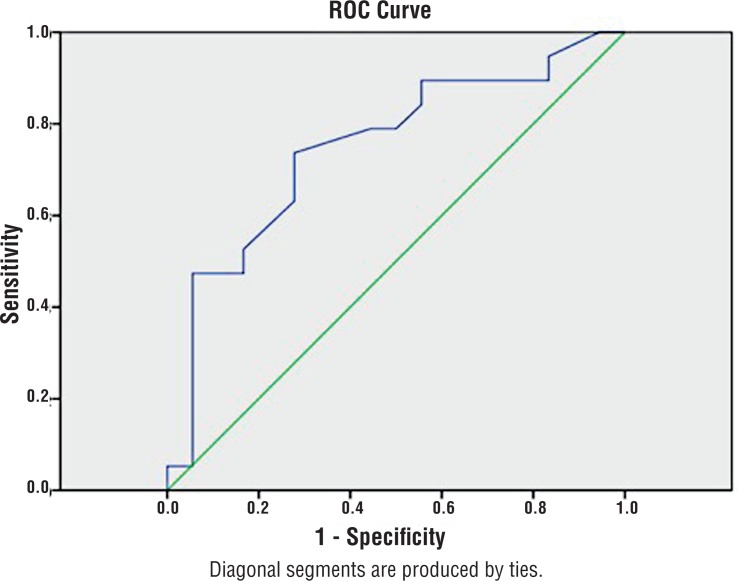
ROC curve analysis. Best correlation with 5.15 cm, sensitivity of 73% and specificity of 72%. AUc = 0.75 (p=0.01).

The ROC curve showed a correlation between the size of diverticula and the risk of AUR. The value of 5.15 cm presented the best correlation with a sensitivity of 73% and a specificity of 72%. The area under the curve (AUC) was 0.75 with p=0.01 (Figure-2).

When we compared patients with BD greater than 5.0 cm with the ones measuring 5.0 cm or less, the median age, prostate weight, PSA and uroflowmetry were similar between groups. The difference was observed in the rate of AUR, with incidence of 27.8% in the group with BD ≤5 cm and 74% in the BD >5 cm group resulting in a OR of 2.65 (1.20-5.85), p=0.005 (Table-2).

**Table 2:** Bladder diverticula ≤5 cm and >5 cm.

	BD ≤5 cm	BD >5 cm	*p*	OR (95%CI)
	Mean±SD	Mean±SD		
Age (years)	71.3 (9.6)	65.6 (11.4)	0.11 a	
Prostate weight (g)	60.3 (55.2)	59.6 (49.0)	0.96 a	
PSA (ng/dL)	8.8 (24.0)*	4.7 (5.6)*	0.49 b	
Qmax pre (mL/s)	6.8 (5.4)	7.3 (6.6)	0.80 a	
Qmax pos (mL/s)	19.8 (7.6)	14.9 (8.7)	0.30 a	
AUR	26%	74%	0.005 c	2.65 (1.20-5.85)

a Student T Test;

b Mann Whitney;

c Chi Square

* median

The pathologic evaluation showed that all BD were pseudodiverticula and 1 case in 47 (2.1%) presented a low grade urothelial carcinoma restricted to the BD, excised with free margins.

## DISCUSSION

The AUR is one of the worst complications of BPH with serious effects in the patient's quality of life. The incidence is approximately 10 per cent over the 70 years and 30 per cent over the 80 years (5, 6). The Proscar Long-Term Efficacy and Safety Study (PLESS) control arm showed that 7% of the patients with BPH followed for four years had AUR (7). Large sample studies show that a prior AUR is the strongest predictor of a new AUR episode (8, 9).

Due to its low incidence, no study so far has included BD as a potential risk factor for AUR in their analysis. The Olmstead County trial found that AUR was related to age, moderate to severe symptoms, a peak flow rate of 12 mL/s and a prostate volume >30 mL (5). Meigs et al. in a study of health professionals found that age >70 years, moderate to severe LUTS and use of medications related to adrenergic or anticholinergic effects were related to an increase risk of AUR (10). Roehrborn et al. correlated the PSA value above 2.5 ng/dL, prostate volume >30 mL and severe LUTS with an increase of AUR (7, 11). None of these studies included the presence of BD in their analysis.

To date, there is no consensus in the literature regarding the role of BD in the prevalence of AUR or even when they should be treated. In the present work, we added some practical information that might help urologist's decisions in the clinical setting. We found that a BD greater than 5.0 cm presents an OR of 2.65 for the occurrence of AUR when compared to patients with BD smaller than 5.0 cm. In the multivariate analysis, only the size of the diverticula reached statistical significance for the development of AUR. The main relevant aspect of this finding is that BD larger than 5.0 cm may have a greater influence in the bladder functioning.

BD may be congenital or acquired. A congenital diverticula involves all layers of the bladder wall, whereas acquired diverticula is always a consequence of obstruction, and is characterized by the herniation of mucosa through the muscle layer of the bladder forming a pseudodiverticula (12). An animal study with an induced rabbit model of diverticula showed that the diverticula reduces the cystometric bladder capacity and compliance besides increasing the residual urine (PVR). There is an increase in the filling detrusor pressures and detrusor thickness (13). There are no studies analyzing the impact of diverticula in bladder function or AUR in men with BPH. Additionally, there is no consensus in literature regarding the size of BD that should be an indication for surgery.

The classic indications for surgery of BD includes presence of tumor, persistent urinary tract infections, bladder stone, hydronephrosis caused by the diverticula, urinary retention with poorly draining diverticula and severe LUTS with paradoxal incontinence (1, 2, 14). A large size with small drainage orifice and diverticula in young man also can be strong indications for surgery. With the development of the minimally invasive techniques, as laparoscopy and robotic, the excision of the BD becomes less morbid with fast recovery and excellent results (15-17).

For the laparoscopic cases of this analysis, we carried out through the extravesical access. In small BD cases, the Collins knife can be used to incise the neck of the diverticula and we prefer this technique for those smaller than 3 cm.

The traditional open method (intra or extravesical) is still very useful mainly with associated procedures as adenomectomies or cystolithotomies and ureteral reimplantation (14, 18). We have been using the intravesical approach for all open cases, because it is easier than the extravesical route and allow us to do associated surgeries with less dissections and complications (19).

To our knowledge, this is the first study analyzing the role of the size of the diverticula as a risk factor for AUR in the context of BPH patients who are candidates to surgery. The clinical aspects and the impact of BD were evaluated in order to stablish a diverticula size cut-off that increase the risk of complications. The clinical setting was AUR, a dramatic complication of BPH.

Limitations of this study are the lack of information regarding preoperative international prostate symptom score (IPSS) and urodynamic data. However, all the patients included in this series were candidates to surgery and were classified into the severe symptoms group. We also did not analyze the occurrence of other complications related to bladder diverticula such as urinary tract infections.

Additionally, we did not analyze patients with BD who were not candidates to surgery. This fact precludes any conclusions regarding the actual role of the BD in the general population with BPH. Further prospective series are needed to accurately analyze the role of BD on the natural history of men with LUTS due to BPH. Finally, we didn't include in this study a control group of patients who were candidates to prostate surgery without BD.

## CONCLUSIONS

The diameter of BD is an independent risk factor for AUR in patients with BPH and BD who are candidates to surgery. A diameter greater than 5.15 cm was related to an increased risk of AUR. This information must be discussed with the patient when considering the risk and benefits of diverticula excision in the context of BPH.

## Abbreviations

AUR: =acute urinary retention
BD: =bladder diverticula
BPH: =benign prostatic hyperplasia
DM: =diabetes mellitus
LUTS: =lower urinary tract symptoms
Qmax: =maximum flow rate
Qmax pre: =preoperative maximum flow rate
Qmax pos: =postoperative maximum flow rate
IPSS: =international prostate symptom score
PVR: =residual urine
PSA: =prostate specific antigen
TURP: =transurethral resection of prostate
UTI: =urinary tract infections
